# Determinants of anticoagulant therapy in atrial fibrillation at discharge from a geriatric ward: cross sectional study

**DOI:** 10.1007/s11239-019-01937-3

**Published:** 2019-08-30

**Authors:** Z. B. Wojszel, A. Kasiukiewicz

**Affiliations:** 1 Department of Geriatrics, Medical University of Bialystok, Fabryczna Str. 27, 15-471 Bialystok, Poland; 2 Department of Geriatrics, Hospital of the Ministry of Interior in Bialystok, Bialystok, Poland

**Keywords:** Atrial fibrillation, New oral anticoagulant drugs, Older people, Comprehensive geriatric assessment, Frailty

## Abstract

Oral anticoagulants (OACs) are effective in preventing stroke in older people with atrial fibrillation (AF), but they are often underused in this particularly high-risk population. The aim of the study was to identify health and functional determinants of oral anticoagulant therapy (OA) in AF at discharge from a geriatric sub-acute ward. A cross-sectional study was conducted and patients who presented with atrial fibrillation were analyzed. They were interviewed, examined, assessed with comprehensive geriatric assessment protocol, and had their hospital records analyzed. Relative risks for OA were counted and multivariable logistic regression model was built. 95 patients took part in the study (22.8% of 416 consecutively admitted to the department, 31.9% men, 73.7% 80 + year-old). 25.8% of them were on antiplatelet drugs and 58.9% on OACs. The percentage on OACs increased significantly to 73.7% at discharge (p = 0.004), mainly due to the new OACs prescription (from 11.8 to 33.3%; p < 0.001). Severe frailty (7 point Clinical Frailty Scale ≥ 6) and anemia presence, but not the risk of bleeding according to the HAS-BLED score, significantly decreased the probability of OACs prescription at discharge. There was also a trend for an association of OACs prescription with the higher total score of CHA2DS2-VASc scale. We conclude that in the real-life population of patients with AF comprehensive geriatric assessment might allow to increase significantly the number of patients on OACs, but it is limited by patient’s frailty status and anemia diagnosis.

## Highlights

- We assessed health and functional determinants of oral anticoagulant (OAC)-prescribing in older patients with non-valvular atrial fibrillation (NVAF) at the sub-acute department of geriatrics.
- Our data revealed the underuse of anticoagulation in older patients with NVAF-only 3/5 of them were taking OACs at admittance.
- Hospitalization had enabled significant improvement in compliance with the NVAF anticoagulant guidelines. This was mainly due to the increase in prescriptions for new OACs, although it was negatively determined by patient’s frailty status and anemia diagnosis.
- Comprehensive geriatric assessment and shared decision making by clinicians, patients and their carers are crucial while establishing treatment plans, and might allow to increase significantly the number of patients discharged with a prescription for OACs.

## Introduction

Atrial fibrillation (AF) is the most common rhythm disorder among older patients. It has a substantial impact on both morbidity and mortality, and is a strong independent risk factor for stroke [1]. Anticoagulant therapy reduces the risk of stroke significantly and can prolong life [2]. Because of that it is recommended that individuals with non-valvular atrial fibrillation (NVAF) should be placed indefinitely on chronic oral anticoagulation.

Although oral anticoagulants (OACs) are effective in preventing stroke in older people with AF, they are often underused in this particularly high-risk population, albeit use of OACs increased over the last decade in Europe [3]. It is assumed that assessing the risk of bleeding should not be the ultimate deciding factor for exclusion from anticoagulation, but rather should support the overall judgment of the patient’s health situation. Prior to considering oral anticoagulant therapy in an older frail patient, a comprehensive geriatric assessment (CGA) should be performed to evaluate its risks and benefits [4]. Available data, regarding clinical profile and management of older patients with AF according to dependency, frailty and cognitive deterioration, are scarce. It is also not known what is the impact of the assessment of these domains on the decision on the recommendation of anticoagulants in the case of patients hospitalized in a geriatric ward.

The aim of the study was to assess the prevalence of oral anticoagulant treatment in patients with NVAF admitted to the geriatric ward, and to determine factors associated with oral anticoagulation at discharge.

## Methods

### Setting, inclusion criteria

We performed secondary analysis of data obtained in the prospective cross-sectional study on frailty syndrome and multimorbidity in patients hospitalized in a geriatric ward. The initial study was conducted in the Department of Geriatrics of the Hospital of the Ministry of Interior in Bialystok, Poland, and all consecutive patients admitted to the department for the first time between 1st September, 2014 and 30th April, 2015 took part in it [5]. The geriatric department is a sub-acute care ward, where older people with multimorbidity and physical and/or cognitive disability are admitted mainly in a planned manner. Patients are referred to the department by GPs, other specialists and from other wards or care facilities. The average waiting time for admission to the ward is approximately 3 months, and the mean length of stay is 7 days. A comprehensive geriatric assessment carried out by a multidisciplinary team, including reviewing and modifying patient’s pharmacotherapy, is one of the goals of hospitalization. The above is intended to reduce polytherapy and identify reasons of patients’ functional decline, malnutrition, recurrent falls or other geriatric syndromes they are suffered from, which are usually multifactorial in nature, and often co-exist with each other.

The study population included all patients with NVAF according to ICD10 (code I48). We did not differentiate between permittent or paroxysmal AF, as there were indications to anticoagulation in both cases. The information about atrial fibrillation was captured based on an interview collected from the patient and from his or her caregiver, verified by a review of all of the patient’s medical records available, by the results of electrocardiogram (ECG), and/or by 24-h ECG monitoring in chosen cases.

### Study design and methods

Stroke prevention both prior to admittance and recommended at discharge was evaluated (use of OACs: vitamin K antagonists—VKAs or new OACs—NOACs, antiplatelet medications—APTs and low-mass-weight heparins—LMWHs). CHA2DS2-VASc and HAS-BLED scores were calculated. On the basis of medical interview with patient and his or her caregiver, followed by thorough clinical examination and review patient’s medical records, we gathered information on patients’ age, gender, place of residence, history of hospitalization and falls in the last 12 months, comorbidities (of 14 chronic diseases: peripheral arterial disease, ischemic heart disease, myocardial infarction, chronic cardiac failure, hypertension, stroke, chronic obstructive pulmonary disease, diabetes/prediabetes, neoplasm, dementia, parkinsonism, chronic arthritis, chronic renal disease, osteoporosis), number of medications taken at admittance, the ability to carry out basic activities of daily life (the Barthel Index [6]), risk of pressure sores (the Norton Scale [7]), risk of falls (the Performance Oriented Mobility Assessment—POMA [8], and Timed Up and Go test—TUG [9]), cognitive abilities (the Abbreviated Mental Test Score—AMTS [10]), risk of malnutrition (the Mini Nutritional Assessment-Short Form—MNA-SF [11]), body mass index—BMI, waist-hip ratio—WHR, albumin level, number of lymphocytes in blood, hemoglobin level and renal function (glomerular filtration rate—GFR, counted using the CKD-EPI formula [12], serum creatinine level). Frailty status was assessed with seven item Canadian Study of Health and Aging Clinical Frailty Scale (CFS) [13]. Hand grip strength of the dominant hand (mean of two measurements) was assessed with a manual hydraulic dynamometer SAEHAN DHD-1. Gait speed was measured during the 4.57 m walk at usual pace and prevalence of orthostatic hypotension was checked.

### Study parameters

Severe frailty was defined as CFS score of 6 or 7. Polypharmacy was defined as 5 or more drugs taken. Multimorbidity was defined as 5 or more diseases of 14 listed. Orthostatic hypotension was diagnosed if systolic pressure decreased by 20 mm Hg or diastolic pressure decreased by 10 mmHg in the first or the third minute of the active standing test. Malnutrition was suspected if albumin level was < 35 g/L, if lymphocytes number was < 1.5 K/L, or if MNA-SF score was below 8. Slowness was diagnosed with cut-off points stratified by gender and height and weakness stratified by gender and BMI quartiles, according to the literature [14].The diagnosis of dementia was based on the thorough neuropsychological examination. Anemia was diagnosed if the hemoglobin level was below 14.0 g/dL in men and below 12.0 g/dL in women. CHA2DS2-VASc score ≥ 2 was interpreted as the high risk of thromboembolic events and HAS-BLED score ≥ 3 as the high risk of bleeding. Chronic kidney disease (CKD) i.e., stage 3, 4 and 5 CKD according to Kidney Disease Outcome Quality Initiative (KDOQI) was diagnosed if GFR was < 60 mL/min/1.73 m^2^. The high risk of falls was diagnosed if POMA score was < 19, and if TUG time was ≥ 14 s.

### Statistical analysis

Data were collected and analyzed using IBM SPSS Version 18 Software suit (SPSS, Chicago, IL, USA), and presented as means and standard deviation for normally distributed, as medians and interquartile range for not normally distributed continuous variables, and the number of cases and percentage for categorical variables. Variables’ distribution was assessed with Shapiro–Wilk tests. Proportions were compared using χ^2^ tests, while the Student’s *t* test for independent samples and Mann–Whitney *U* test were used to compare means and medians. To assess differences between two dependent variables, Wilcoxon signed-rank test was used. Relative risks (*RR*s) were calculated to evaluate the potential risk factors that might influence a decision about oral anticoagulation. It was followed by a multivariable logistic regression analysis including predictors with a *p* value of *RR* less than 0.1, and excluding those highly correlated to avoid multicollinearity. We reported ORs with 95% CIs and *p* values for each model parameter. A two-tailed *p* value of less than 0.05 was regarded as significant. Missing values were omitted and statistics were calculated for the adequately reduced groups.

## Results

**Fig. 1:**
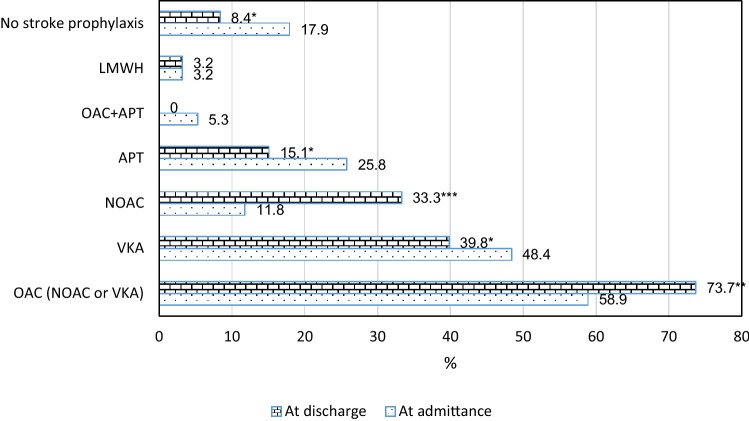
Stroke prophylaxis at admittance and at discharge from the geriatric ward. *APT* antiplatelet medication, *LMWH* low-molecular-weight heparin, *NOAC* new oral anticoagulant, *OAC* oral anticoagulant, *VKA* vitamin K antagonist; *p < 0.05; **p < 0.01; ***p < 0.001 (Wilcoxon test, not available for variables with low number of cases). APT at admission: aspirin (ASA)—22 cases; dual APT (ASA + clopidogrel)—2 cases. APT at discharge: ASA—11 cases; clopidogrel—3 cases; dual APT—1 case

98 of 416 patients hospitalized in the study period were diagnosed with AF. 95 (22.8%) were included in the analysis (two patients died and one was transferred to another department). The majority of them was above 80 years of age (73.7%), and female (78.1%). 25.8% of the study group were on APTs and 58.9% on OACs, mainly on VKAs (Fig. 1). The percentage on OACs increased significantly to 73.7% at discharge (p = 0.004), mainly due to the increase of prescriptions for NOACs.

**Fig. 2:**
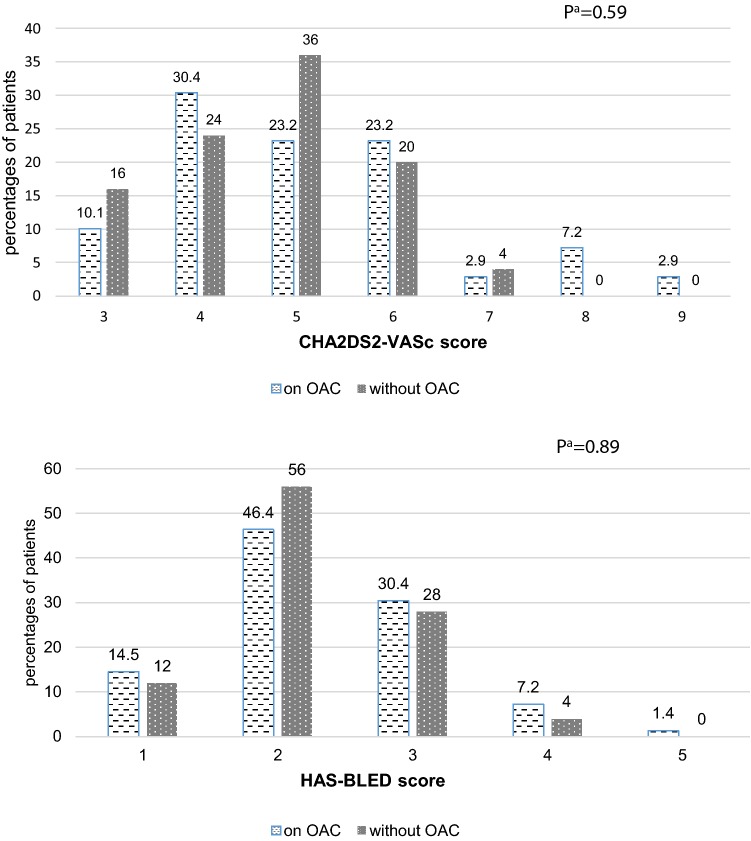
CHA2DS2-VASc and HAS-BLED scores distribution in patients with atrial fibrillation on OACs and without OACs at discharge. *OAC* oral anticoagulant; ^a^χ^2^ test

Patients prescribed and not prescribed OACs at discharge did not differ in age, gender and in the majority of health characteristics analyzed, although they differed significantly in the median value of CFS, in GFR and in hemoglobin level and in the prevalence of pressure sores (Table 1). Severe frailty, Barthel Index < 80 points and albumin level below 3.5 g/L were negatively associated with OACs use at discharge. The groups did not differ in median values of CHA2DS2-VASc and HAS-BLED scales, and in distribution of their scores (Fig. 2). The lowest CHA2DS2-Vasc score was 3, so the whole study group had the indication for anticoagulation.

**Table 1:** Characteristics of study groups

Parameter	Total	Missing values	Patients on OAC	Patients without OAC	*p* value^a^	*RR* (*CI*)
No. (%) of patients	95 (100.0)		70 (73.7)	25 (26.3)		
Age, years, M (SD)	83.14 (5.7)	–	82.8 (6.0)	84.2 (4.4)	0.29	
Age, 80 +, n (%)	70 (73.7)	–	50 (71.4)	20 (80.0)	0.40	0.89 (0.70–1.14)
Gender, men, n(%)	30 (31.9)	–	23 (32.9)	7 (28.0)	0.65	1.06 (0.82–1.37)
Place of residence, rural, n(%)	19 (20.0)	–	14 (20.0)	5 (20.0)	1.0	0.92 (0.66–1.28)
Number of chronic diseases^b^, Me (IQR)	5 (4–7)	–	5 (4–7)	5 (4–6)	0.29	
Multimorbidity, n (%)	63 (66.3)	–	48 (68.6)	15 (60.0)	0.44	1.11 (0.85–1.45)
Number of drugs at admittance, M (SD)	8.5 (3.2)	2	8.4 (3.0)	8.8 (3.6)	0.67	
Polypharmacy, n (%)	85 (91.4)	2	63 (92.6)	22 (88.0)	0.48	1.19 (0.68–2.06)
Hospitalization in the last 12 months, n (%)	35 (37.2)	1	28 (40.6)	7 (28.0)	0.27	1.15 (0.91–1.46)
Cardiovascular diseases						
Ischemic heart disease, n (%)	56^b^ (58.9)	–	44 (62.9)	12 (48.0)	0.20	1.17 (0.89–1.53)
Myocardial infarction, n (%)	12 (12.6)	–	9 (12.9)	3 (12.0)	0.91	0.89 (0.59–1.34)
Hypertension, n (%)	78 (82.1)	–	57 (81.4)	21 (84.0)	0.77	0.99 (0.72–1.36
Chronic cardiac failure, n (%)	69 (72.6)	–	52 (74.3)	17 (68.0)	0.55	1.10 (0.81–1.49)
Peripheral arterial disease, n (%)	27 (28.4)	**–**	25 (35.7)	2 (8.0)	0.008	1.46 (1.19–1.80)
Stroke/TIA, n (%)	20 (21.1)	–	15 (21.4)	5 (20.0)	0.88	1.02 (0.77–1.36)
Diabetes, n (%)	38 (40.0)	–	30 (42.9)	8 (32.0)	0.34	1.18 (0.93–1.51)
Dementia, n (%)	34 (35.8)	–	27 (38.6)	7 (28.0)	0.34	1.13 (0.88–1.45)
Neoplasm, n (%)	6 (6.3)	–	3 (4.3)	3 (12.0)	0.17	0.58 (0.25–1.38)
HAS-BLED scale, Me (IQR)	2 (2–3)	–	2 (2–3)	2 (2–3)	0.64	
HAS-BLED ≥ 3, n (%)	35 (37.2)	1	27 (39.1)	8 (32.0)	0.53	1.08 (0.85–1.38)
CHA2DS2-VASc scale, Me (IQR)	5 (4–6)	1	5 (4–6)	5 (4–5.5)	0.39	
Hemoglobin, g/dL, M (SD)	12.2 (1.9)	2	12.6 (1.8)	11.6 (1.9)	0.03	
Anemia, n (%)	52 (55.0)	2	34 (50.0)	18 (72.0)	0.06	0.78 (0.62–1.0)
GFR, ml/min/1.73 m^2^, M (SD)	51.7 (17.8)	2	54.1 (17.8)	44.8 (16.3)	0.03	
GFR < 60 ml/min./1.73 m^2^, n (%)	63 (66.3)	2	44 (62.9)	19 (76.0)	0.23	1.87 (0.66–5.28)
Serum creatinine, mg/dL, M (SD)	1.19 (0.38)	2	1.14 (0.36)	1.32 (0.41)	0.05	
BMI, kg/m^2^, M (SD)	30.4 (5.5)	19	30.4 (5.38)	30.1 (5.9)	0.83	
WHR, m, M (SD)	0.93 (0.1)	10	0.94 (0.1)	0.92 (0.1)	0.45	
Albumin, g/L, M (SD)	35.3 (3.5)	2	38.1 (4.6)	38.2 (3.1)	0.89	
Albumin < 35 g/L, n (%)	14 (15.1)	2	7 (10.1)	7 (29.2)	0.03	0.64 (0.37–1.09)
Lymphocytes, K/μL, M (SD)	1.6 (0.6)	3	1.6 (0.7)	1.5 (0.5)	0.75	
Lymphocytes < 1.5 K/μL, n (%)	47 (51.1)	3	34 (50.7)	13 (52.0)	0.92	0.99 (0.77–1.27)
MNA-SF, Me (IQR)	11 (9–13)	4	11 (10–13)	11 (5–13)	0.29	
MNA-SF score < 8, n (%)	14 (15.4)	4	8 (11.8)	6 (26.1)	0.10	0.73 (0.46–1.17)
CC, cm, M(SD)	35.4 (5.4)	9	35.6 (5.2)	34.9 (6.1)	0.66	
CC < 31 cm, n (%)	17 (19.8)	9	11 (16.9)	6 (28.6)	0.24	0.83 (0.57–1.20)
MAC, cm, M(SD)	28.6 (3.9)	8	29.0 (3.9)	27.5 (3.5)	0.12	
MAC ≤ 22 cm, n (%)	15 (17.2)	8	9 (13.8)	6 (27.3)	0.15	0.77 (0.50–1.19)
Handgrip strength, kg, M (SD)	19.6 (8.4)	18	19.4 (8.0)	20.1 (9.9)	0.77	
Weakness, n (%)	52 (67.5)	18	40 (67.8)	12 (66.7)	0.93	1.01 (0.78–1.32)
Norton scale, Me (IQR)	18 (15–19)	–	18 (16–19)	16 (12–19)	0.48	
Norton scale score < 14, n (%)	22 (23.2)	–	13 (18.6)	9 (36.0)	0.08	0.76 (0.52–1.09)
Pressure sores, n(%)	9 (9.7)	2	4 (5.9)	5 (20.0)	0.04	0.58 (0.28–1.22)
Orthostatic hypotension, n (%)	12 (15.8)	19	9 (14.5)	3 (21.4)	0.599	
Falls in the last 12 months, n (%)	35 (44.3)	16	29 (49.2)	6 (30.0)	0.14	1.22 (0.95–1.56)
POMA, M (SD)	21.2 (6.5)	27	21.1 (6.8)	21.3 (5.7)	0.96	
POMA < 19, n (%) N	20 (29.4)	27	16 (28.6)	4 (33.3)	0.74	0.96 (0.75–1.24)
TUG, s, M (SD)	23.8 (13.9)	28	23.2 (14.2)	26.0 (13.2)	0.51	
TUG ≥ 14 s, n (%)	49 (73.1)	28	39 (72.2)	10 (76.9)	0.73	0.96 (0.74–1.23)
Gait speed, m/s, M (SD)	2.0 (0.8)	29	1.9 (1.0)	2.2 (1.4)	0.69	
Slowness, n (%)	40 (60.6)	29	33 (62.3)	7 (53.8)	0.58	1.07 (0.83–1.38)
Barthel Index, Me (IQR)	90 (65–100)	–	90 (75–100)	85 (35–95)	0.13	
Barthel Index < 80, n (%)	29 (30.5)	–	18 (25.7)	11 (44.0)	0.09	0.79 (0.58–1.08)
AMTS, Me (IQR)	8 (5–9)	9	8 (5.75–9)	7. (5–9)	0.48	
AMTS < 7, n (%)	29 (33.7)	9	22 (33.3)	7 (35.0)	0.89	0.98 (0.77–1.26)
Dementia, n (%)	34 (35.8)	–	27 (38.6)	7 (28.0)	0.34	1.13 (0.89–1.43)
CFS, Me (IQR)	5 (4–6)	1	5 (4–5)	5.5 (4.5–6.5)	0.05	
Severe frailty, n (%)	28 (29.5)	–	16 (22.9)	12 (48.0)	0.02	0.71 (0.50–0.99)

^a^χ^2^ test or Fisher exact test, as appropriate, for categorical variables; Mann–Whitney test or *t*-test for interval variables

^b^14 patients had a history of prior percutaneous coronary intervention (PCI) with stents, one—of prior coronary artery bypass graft (CABG), and one—of prior CABG and PCI; in 3 cases it was PCI with stents in the last 12 months before hospitalization

*AF* atrial fibrillation, *AMTS* abbreviated mental test score, *BMI* body mass index, *CC* calf circumference, *CFS* 7-point clinical frailty scale, *GFR* glomerular filtration rate, *IQR* interquartile range, *M* mean value, *MAC* mid-arm circumference, *Me* median value, *MNA-SF* Mini Nutritional Assessment-Short Form, *n* number of cases, *POMA* Performance Oriented Mobility Assessment, *TIA* transient ischemic attack, *TUG* Timed Up and Go test, *SD* standard deviation, *WHR* waist-hip ratio

A direct multivariable logistic regression analysis was performed on OACs prescription at discharge as outcome and five predictors: severe frailty, albumin < 35 g/L, anemia, HAS-BLED score ≥ 3, CHA2DS2-VASc total score (Table 2). Among variables meeting the criterion p < 0.1 severe frailty, risk of pressure sores according to the Norton scale and their prevalence, dependency in ADL (Barthel Index < 80 points) and risk of malnutrition according to MNA-SF were strongly correlated with each other. Therefore, to avoid multicollinearity, only the variable “severe frailty”—that could be a construct representing the remaining ones—were included in the model. Serum creatinine and GFR correlated strongly with anemia and peripheral arterial disease correlated strongly with CHA2DS2-VASC score. So we have decided not to include serum creatinine, GFR and peripheral arterial disease in the final logistic regression model. We adjusted for the CHA2DS2-VASc score and HAS-BLED score ≥ 3 irrespectively of associations in bivariate analyses. Prediction success was acceptable, with 92.9% of the OACs prescription (sensitivity) and 41.7% of no OACs prescription (specificity) correctly predicted, for an overall success rate of 78.9%. An independent negative effect associated with the prescription of OAC was observed for severe frailty (odds ratio, 0.27; 95% CI 0.08–0.94; p = 0.04) and anemia (odds ratio, 0.25; 95% CI 0.07–0.86; p = 0.03); there was also a trend for an association with the higher CHA2DS2-VASc (odds ratio, 1.63; 95% CI 1.00–2.67; p = 0.05), when controlling for albumin level below 35 g/L and HAS-BLED score ≥ 3.

**Table 2:** Multivariable logistic regression analysis for OACs recommended at discharge

	OR	95% CI	*p* values
Severe frailty (CFS 6 and 7)	0.27	0.08–0.94	0.04
Anemia	0.25	0.07–0.86	0.03
Albumin < 35 g/L	0.37	0.09–1.60	0.18
CHA2DS2-VASc score	1.63	1.00–2.67	0.05
HAS-BLED score ≥ 3	2.73	0.73–10.25	0.14

*OR* odds ratio, *CFS* the 7-point Clinical Frailty Scale, *CI* confidence interval

We looked more in depth into the cases without OACs at discharge. In two cases the acute bleeding was diagnosed. Medical records of the next ten patients contained information about labile INR (or inability to control INR, mainly due to disability) and refusal to receive NOAC. Two of three patients discharged on LMWHs were transferred to Palliative Care Unit because of the advanced neoplasms; the last one had very low GFR (18.6 mL/min/1.73 m^2^) and no possibility for regular control of INR. In case of eight patients who received no anticoagulant nor antiplatelet therapy at discharge we observed anemia and the average level of hemoglobin in this group was significantly lower than in the group of patients who were recommended OACs, APTs or LMWHs at discharge (10.13 ± 2.13 g/dL and 12.49 ± 1.76 g/dL respectively, *p* = 0.001). This group was significantly more likely to have low albumin level, severe frailty syndrome, had the lowest eGFR and Barthel Index scores compared to those who received OACs, APTs or LMWHs. The reason for the lack of anticoagulation at discharge in two last cases was not obvious.

## Discussion

Albeit the net clinical benefit of anticoagulant treatment even in very old patients with NVAF is positive, a large proportion of older patients are not treated with OACs [15–17]. It was confirmed also by our study—41.1% of patients with NVAF admitted to the geriatric department did not receive OACs. The results were similar to those obtained by Ekerstad et all in acutely hospitalized frail patients over the age of 75 years—the prevalence of AF was 47%, and only 63% of patients were prescribed an anticoagulant [18].

Anticoagulation therapy in older patients is often a challenge, because of the frequent high risk of both stroke and bleeding in this population. In our study all of the participants had the risk of stroke assessed with CHA2DS2-VASc scale suggesting the need for oral anticoagulation (the score ≥ 2), and at the same time 37.2% of the group had the high risk of bleeding (HAS-BLED score ≥ 3). Despite that, there was a possibility to increase significantly the usage of oral anticoagulants up to 73.7% at discharge (p = 0.004), as there was no documented absolute contraindications to OACs. This problem can affect almost half of geriatric wards patients [18]. So hospitalization in a geriatric ward represents a double opportunity, allowing not only to review and update the patterns of therapy, but also to include in an anticoagulant scheme therapy subjects who were previously considered unsuitable because of adherence or safety issues [19].

The improved safety profile of NOACs may enable treatment of older patients that were previously untreated. NOACs showed better efficacy and equivalent safety compared to warfarin even in those with moderately impaired renal function [20]. The increase in oral anticoagulant therapy in our study was mainly due to an increase in the NOAC prescription (from 11.8 to 33.3%; p < 0.001). The prescription of NOACs has increased, and use of VKAs has dropped significantly in many countries [21–23], and a progressive increase in the proportion of patients newly diagnosed with AF receiving guideline-recommended therapy, potentially driven by the availability of NOACs, was also observed [24–27]. But in the period that was covered by our study (and it is also the case now) all NOACs in Poland were not refunded in AF, and the price of these medications was quite high. The change of health insurance coverage policy could substantially influence OACs prescription pattern in Poland, as it was observed in other countries [28]. The similar situation is in the Canadian universal healthcare system, that covers the cost of NOACs for select patient groups. In the study performed in Ontario, NOAC users had a higher median neighborhood income than VKA users, reported higher annual household income, and patients with private insurance were more likely to use NOACs than those without insurance [29].

Our results confirmed that APTs were often inappropriately prescribed instead of OACs—25.5% of patients with AF were on APTs at admittance, but this percentage dropped significantly to 15.1% at discharge (p = 0.02). The underuse of OACs in other studies was often associated with the prescription of APTs in older patients with AF, regardless of the presence or absence of known atheromatous disease [30]. But we have to underline, that according to the 2012 ESC Guidelines—in force during the period of the study—the use of antiplatelet therapy for stroke prevention in AF could had been recommended in case of patients who refused any form of oral anticoagulation [31].

Our data did not confirm that lack of prescription for OAC at discharge was due to physicians’ fear of bleeding, and neglecting the thromboembolic risk. In multivariable logistic regression analysis a trend for an association of OACs prescription with the higher total score of CHA2DS2-VASc scale was noticed, whereas the HAS-BLED score did not correlate negatively with that. Contrary to Diez-Manglano and co-workers findings [32], OACs were recommended significantly more frequently in patients with peripheral artery disease in our study, but other cardiovascular diseases were not connected with the OACs prescription at discharge.

Severe frailty was observed in 29.5% of the study group, significantly more frequently in the no-OAC group, and severe frailty and anemia were the independent negative determinants of OAC prescription at discharge when adjusted for albumin level < 35 g/L, HAS-BLED ≥ 3 and CHA2DS2-VASc score. A systematic review on frailty in hospitalized older patients with AF and the use of oral anticoagulation confirmed, that the prevalence of frailty syndrome is high, and frail older patients are significantly less likely to receive OACs [33]. Older patients’ treatment, especially in the advanced age, is generally complicated by many concomitant factors, including adherence, cognitive deterioration, multimorbidity, requiring multiple concomitant medications (with an increased risk of drug interactions and adverse effects), renal impairment, risk of falling, involvement of caregivers, and patient-physician relationship. Underuse of OACs is almost never ascribable to a single geriatric condition or factor, but rather to a combination of barriers [34]. We generally agree with the opinion expressed by Pati and colleagues, that despite that logical considerations and evidence-base data related to the reduced bleeding risk of NOACs make these drugs the anticoagulant agents of choice in frail patients, in the setting of the frail older patients an individualized approach should be taken, taking into consideration the risk of thromboembolic and bleeding events, other comorbidities and patient-related factors, rather than a generalized “one drug fits all” approach [35]. Also each NOAC comes with its own unique advantages and safety profile, so a personalized case by case approach should be adopted to decide on the appropriate anticoagulation regimen for older patients after weighing the overall risks and benefits of therapy [36, 37]. Besides, according to some observations, NOAC prescribing in older patients with NVAF frequently fell short of quality standards, and interventions to enhance the quality of NOAC prescribing in this high-risk population are needed [38, 39].

The strength of our study is that it included very frail older patients with a large disability burden, patients who usually were excluded from most clinical trials. The study was not based on administrative claims data—the health and functional assessment performed within comprehensive geriatric assessment was multidimensional, allowing for the more in-depth analysis of health and functional determinants of anticoagulant therapy.

The study has got some limitations, which should be mentioned. First of all, it was performed not in a sample randomly selected from the general population of older people, so the results can be generalized for the patients of the similar settings only. The study sample was mid-sized, and a larger number of patients would have strengthened the results. As it was a secondary analysis of data previously collected some limitations resulted from that (some data was not available, as indicated in tables; classification of AF was not listed nor was the duration of disease; patient’s/carer’s and provider’s preferences were not fully captured; we were not able to attribute treatment decisions directly to the presence or absence of measured contraindications).

## Conclusions

Our data revealed the underuse of anticoagulation in older patients with AF admitted to the geriatric ward—only 3/5 of them were taking OACs at admittance. Hospitalization had enabled significant improvement in compliance with the NVAF anticoagulant guidelines, and this was mainly due to the increase in prescriptions for NOACs, although it was limited by patient’s frailty status and anemia diagnosis.

## Data Availability

The datasets analysed during the current study are available in the RepOD repository, [http://dx.doi.org/10.18150/repod.5725417].
